# The Relationship of Insufficient Effort Responding and Response Styles: An Online Experiment

**DOI:** 10.3389/fpsyg.2021.784375

**Published:** 2022-01-12

**Authors:** Gene M. Alarcon, Michael A. Lee

**Affiliations:** ^1^Air Force Research Laboratory, Dayton, OH, United States; ^2^General Dynamics Information Technology, Inc., Atlanta, GA, United States

**Keywords:** IRT (item response theory), IRTree, response style, careless responding, insufficient effort responding (IER)

## Abstract

While self-report data is a staple of modern psychological studies, they rely on participants accurately self-reporting. Two constructs that impede accurate results are insufficient effort responding (IER) and response styles. These constructs share conceptual underpinnings and both utilized to reduce cognitive effort when responding to self-report scales. Little research has extensively explored the relationship of the two constructs. The current study explored the relationship of the two constructs across even-point and odd-point scales, as well as before and after data cleaning procedures. We utilized IRTrees, a statistical method for modeling response styles, to examine the relationship between IER and response styles. To capture the wide range of IER metrics available, we employed several forms of IER assessment in our analyses and generated IER factors based on the type of IER being detected. Our results indicated an overall modest relationship between IER and response styles, which varied depending on the type of IER metric being considered or type of scale being evaluated. As expected, data cleaning also changed the relationships of some of the variables. We posit the difference between the constructs may be the degree of cognitive effort participants are willing to expend. Future research and applications are discussed.

## Introduction

The importance of self-report scales to the psychological and sociological sciences cannot be understated. Ideally, self-report scales allow researchers to access psychological processes (personality, attitudes, emotions, etc.) of a large amount of people in a short time. As Spector (2006) noted, an individual is often the person with the most accurate sense of their own psychological processes. However, utilizing self-report scales comes with inherent limitations. Most notably, for researchers to accurately examine psychological constructs with self-report scales, participants must provide accurate responses. Two types of behaviors noted in the literature that may influence the accuracy of self-reports are insufficient effort responding (IER) and response styles. It is problematic when participants respond carelessly or with a specific response style as these can influence the measurement of the underlying factors in numerous ways (Weijters et al., 2010; Van Vaerenbergh and Thomas, 2013; Maniaci and Rogge, 2014). A variety of IER metrics exist in the literature that attempt to assess it across different parameters. Similarly, there are a variety of metrics for understanding response styles in the literature (Van Vaerenbergh and Thomas, 2013). Recent advances in the statistical detection of response styles include a method of modeling known as IRTrees, which postulates that participants respond to survey items using a hierarchical decision process (De Boeck and Partchev, 2012; Böckenholt, 2013). This contrasts with traditional theory of survey respondents viewing scales in an ordinal fashion (Embretson and Reise, 2013). Previous research on IRTrees has noted they fit the data better than traditional ordinal item response theory (IRT) models and they more accurately assess latent traits as they have partialed out the variance due to extreme and midpoint response styles (Böckenholt, 2017; LaHuis et al., 2019). Interestingly, the research on IRTrees and response styles has often failed to explore the influence of IER on the overall model fit and the latent traits derived from the IRTree models. Given that IER and response styles are both constructs that can influence self-reports (Meade and Craig, 2012; Van Vaerenbergh and Thomas, 2013), this is a clear gap in the literature that this paper seeks to address by examining the relationship between the two constructs.

### Insufficient Effort Responding

Participants who pay insufficient attention to the item content are problematic for researchers, as they do not provide quality data. This type of participant behavior has gone by several monikers in the literature such as IER (Huang et al., 2012), inattentive responding (Maniaci and Rogge, 2014), or careless responding (Barnette, 1999; Huang et al., 2012; Bowling et al., 2016). For clarity we refer to the behavior and underlying construct as IER for the rest of the paper. Researchers have theorized IER is due to a lack of motivation, attention, or both (Weijters et al., 2013). Participants lacking motivation may not want to expend cognitive effort on reading and responding to items in a deliberate manner. Similarly, when participants are inattentive due to distractions or carelessness, they may answer at random or with response patterns that require less cognitive resources. Regardless, participants that partake in IER respond to survey items without fully processing the information presented within instructions nor the items they complete for a portion or the entire length of a survey.

The construct of IER is multifaceted and is comprised of random and non-random responding, based on the patterns of IER that a respondent displays (Maniaci and Rogge, 2014). Random responding indicates participants are arbitrarily responding to survey items without thought or intention (Curran, 2016). In contrast, non-random responding indicates the participants are using a response pattern to respond to items in a similar way. For example, a participant endorsing a “4” consistently across items on a 5-point Likert scale is responding with insufficient effort but there is a consistency to the pattern of responses. While both forms can negatively impact data quality, non-random response patterns are more problematic for assessing constructs for two reasons. First, non-random responding can influence data quality, correlations, factor structures and statistical power (Huang et al., 2012; Maniaci and Rogge, 2014). Second, IER interacts with survey type to create correlation bias. In scales without reverse-scored items, IER can inflate the correlation between the constructs that are positively related to each other and deflate the correlation of constructs negatively related to each other. Although some researchers have hypothesized the inflation and deflation of counterbalanced items may offset each other (Spector, 1992), Kam and Meyer (2015) found scales that are counter balanced with positive and negative items had similar effects as scales that were not counterbalanced. As such, it is important to assess IER and remove data from participants that perform IER.

Insufficient effort responding can be assessed via two general methods: direct or indirect. Direct assessments of IER include metrics that assess the construct using unmediated measures, such as page time and attention check items. Page time indices provide a metric of how much time the participants spent on a survey page during online data collection. Participants that spend too little time on a page (e.g., average 2 s per item, per Huang et al., 2012) are flagged as careless responders on the assumption that they did not take adequate time to read, process, and respond thoughtfully to each item. Attention check items ask participants to choose a specific response (e.g., “Please select Strongly Disagree”) or have content so inane or unlikely that a participant would not reasonably endorse it (e.g., “I eat rocks”). Although these direct measures have high face and construct validity, they do not provide information on whether a participant is engaging in IER response patterns across the full length of the survey beyond the check items themselves nor what kind of IER (i.e., random or non-random) the participant is engaging in. To this end, indirect assessments detect patterns or involve statistical analyses to determine if the participant is engaging in IER per scale presented or across the entire survey (Curran, 2016). Depending on the metrics being used, they can detect random or non-random patterns of IER. However, given that there are a wide range of indirect metrics each with their own strengths and limitations, it is often up to the discretion of the researcher to determine which metric is appropriate for use with their data and study design, if any (Curran, 2016).

Another issue with indirect measures is that they may flag participants that are not performing IER. For example, the IER metric of Mahalanobis distance (Mahalanobis D) flags participants as multivariate outliers based on preestablished cutoffs. However, these participants may be responding earnestly and are outliers given their genuine responses. Meade and Craig (2012) note that both direct and indirect measures of IER should be utilized in data cleaning to identify the presence and type of IER that may occur in a data set. Each specific metric can derive a different type of data from sample responses, which can contribute to a holistic determination as to whether a participant has engaged in IER.

### Response Styles

Response styles (also known as response bias) are systematic tendencies of a participant to respond to a range of items on a different basis from what the items are designed to measure (Paulhus, 1991). A variety of response styles have been identified in the literature (for a full review, see Van Vaerenbergh and Thomas, 2013), but we focus on two for the current study: midpoint response style (MRS) and extreme response style (ERS). MRS is the tendency to endorse the midpoint of a scale; this is found in odd-point scales (e.g., 5-point scales) as even-point scales (e.g., 6-point scales; also known as symmetrical scales) do not have a midpoint. ERS is the tendency of the participant to choose the extreme ends of the scale. For example, on a 5-point Likert scale a participant high in ERS will tend to choose the “1” and “5” options.

A well-established tenet of psychometrics is that observed scores are comprised of true variance and error variance (Spector, 1992). Response styles are considered a nuisance variable and part of the error variance, as they are external to the item content and construct being measured. This is not without merit, as response styles can affect the measurement of the underlying latent construct. First, response styles can influence the univariate distributions of the scales (Baumgartner and Steenkamp, 2001), affecting the observed variances and means of the scale. Second, response styles can influence the multivariate distributions, which can carry implications for a range of analyses including normality tests and outlier identification (Tabachnick et al., 2007). Additionally, response styles can reduce the fit of IRT models, as the models violate local independence, which we describe below. As with IER, response styles have the potential to create systematic error for a sample, biasing results and skewing interpretation.

### Insufficient Effort Responding and Response Styles

Several major parallels and comparisons can be drawn between IER and responses styles. They both deal with motivations of the participant. Individuals low in motivation are incentivized to optimize their response patterns so they expend as little energy as possible while answering (Krosnick, 1991). When a participant employs a response style, they may or may not still expend effort by superficially examining items, such as looking at the connotation of the items (e.g., positive or negative) to determine the response set (Van Vaerenbergh and Thomas, 2013). In contrast, the participant does not even superficially examine the items for responses with IER, instead they respond without regard to the item content (e.g., random responding, responding with the same endorsement for all items).

Second, while IER is always driven by a lack of motivation, certain response styles may occur because of motivation to present oneself in a positive light. For example, ERS have been associated with social desirability (Grau et al., 2019), which is the tendency to respond to be viewed as favorable by others (Krumpal, 2013). Although this type of response style obfuscates the construct in question because of motivations to present oneself in a particular manner, they have differential relationships with the construct being measured and IER. As such, response styles and IER have been found to contribute separately to error variance in the observed scores (Grau et al., 2019).

Third, participants performing IER, specifically non-random IER, may respond in a way similar to other response styles. Non-random IER is typically assessed by long-string analysis, which is when a participant responds the same across items, for example responding to every item with a “4.” This type of non-random responding emulates response styles, as researchers could easily mistake a participant performing non-random IER with straight-lining as a response style in a scale with no reverse coded items. Indeed, researchers have advised including reverse scored items to alleviate this concern (Spector, 1992), which Kam and Meyer (2015) note can help with detection but is not infallible. Grau et al. (2019) found non-random IER was positively associated with ERS and negatively associated with MRS in a multicultural study. Interestingly, they also found random IER was positively associated with MRS and ERS. Kam and Meyer (2015) also found participants performing IER were more likely to give identical responses to consecutive survey questions. This research indicates there may be a relationship between response styles and IER, but little research has explored this relationship in a systematic manner.

### IRTrees

Several methods exist for examining response styles such as averages of responses, latent class analyses, the Response Style Questionnaire, and others (for full review see Van Vaerenbergh and Thomas, 2013). Recently, researchers have employed various IRT models to evaluate the impact of response styles on scale scores (Jin and Wang, 2014; see Bolt et al., 2014). A particular type of model that has seen an increase in the literature is the IRTree model (Böckenholt, 2013). The IRTree methodology suggests participants do not respond in a traditional ordinal fashion to Likert type scale, but rather through a sequence of decision processes (De Boeck and Partchev, 2012; Böckenholt, 2013). IRTree models posit participants use a decision hierarchy when responding to items and hypothesize several latent abilities for any scale based on this hierarchy (Böckenholt, 2017).

**FIGURE 1:**
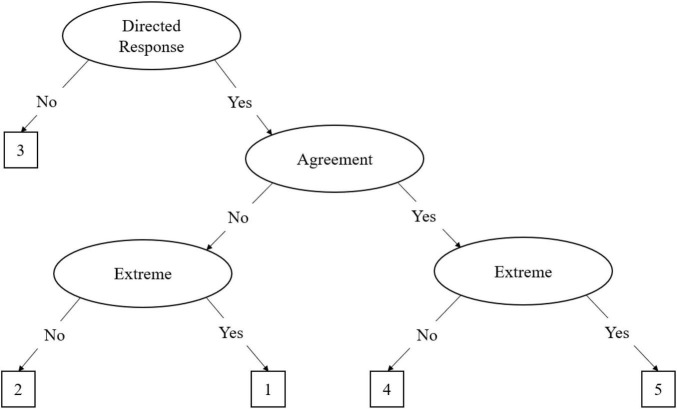
The midpoint primary process (MPP) IRTree decision model. This figure presents the MPP decision hierarchy on a five-point Likert scale ranging from one to five. The process begins with the decision to provide a directed response or not. Not giving a directed response results in the selection of option three and termination of the process; otherwise, the process continues. Once the respondent has determined they will give a directed response, they must determine whether they agree or disagree with the item, then whether it is an extreme agreement or disagreement, selecting the corresponding option on the scale based on where they fall.

IRTree models consist of nodes that define the decision processes, which differ according to the type of Likert-type response scale utilized. Scales that utilize an odd-point scale have a middle response option, and thus typically conform to the midpoint primary process model (MPP; LaHuis et al., 2019). The MPP for a 5-point Likert scale is defined by three pseudo-items which outline the decision nodes, which is illustrated in Figure 1. The first step of the MPP is a decision of a directed response or a neutral response, represented by the midpoint node (θ_M_). If a directed response is chosen, where the participant either agrees or disagrees with the item, the participant continues to the next decision process. However, if the participant chooses a neutral response the decision hierarchy is terminated. The second step is the decision to agree or disagree with the item, which is represented by the agreement node (θ_A_). The third step is a decision to endorse an extreme response, given the directed response (θ_E_). In other words, if the participant chose an agreement directed response, the extreme response node is the decision to endorse the extreme agreement or not. Although this pattern refers to a 5-point Likert scale, the decision processes can be extrapolated to other odd-point scales, such as the 7-point Likert scale, with additional decision processes.

**FIGURE 2:**
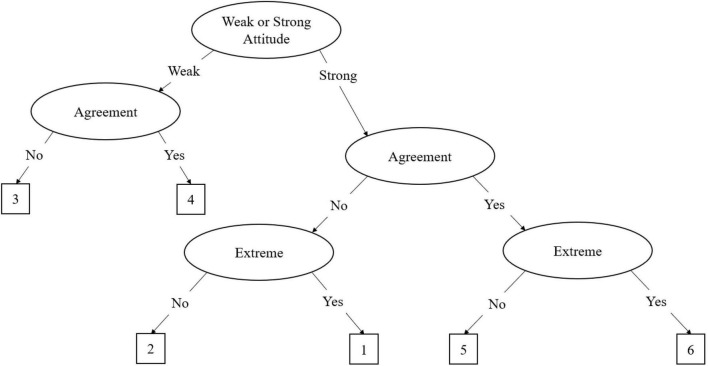
The agreement primary process (APP) IRTree decision model for six-point scales. In this figure, the six-point Likert scale ranges from one to six. The process begins with the decision regarding whether the respondent has a strong or weak attitude toward the item. Regardless of their decision at this node, the respondent must subsequently decide whether they agree or disagree with the item. If they have a weak attitude, the process ends at the weak agreement node, selecting options three or four. If the respondent has a strong attitude, they must subsequently decide whether they have an extreme agreement or disagreement, then select the corresponding scale option.

**FIGURE 3:**
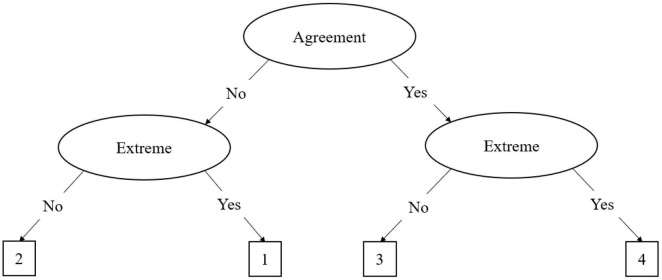
The agreement primary process (APP) IRTree decision model for 4-point scales. In this figure, the 4-point Likert scale ranges from one to four. The process begins with the decision regarding whether the respondent agrees or disagrees with the item. Then, they must subsequently decide whether they have an extreme agreement or disagreement, then select the corresponding scale option.

The agreement primary process (APP) model is similar to the MPP model, but it applies to even-point scales. In even point scales there is no true middle as in odd-point scales; as such, the model is adjusted slightly. Böckenholt (2013) described the decision tree for a 6-item even-point response scale, which is illustrated in Figure 2. In this tree, the first decision node is whether the participant has a strong (θ_S_) or weak attitude (θ_W_). As the APP model does not have a midpoint, the model treats the middle two response options as weak attitude responses, which can be delineated into weak agreement (θ_WA_) or weak disagreement (θ_WD_). Like the midpoint in the MPP, the process terminates once weak agreement or disagreement has been determined. Should the participant have a stronger attitude, the second process determines whether the participant agrees or disagrees with the item. In this second process, agreement (θ_A_) and disagreement (θ_D_) are differentiated. The final process determines the extremity of the agreement or disagreement with the item. A similar method is discussed by Meiser et al. (2019) for applying the APP model to 4-point Likert scales. As illustrated in Figure 3, the 4-point MPP model eliminates the weak agreement node, leaving just the agreement and extreme nodes. Variations of these models can be applied for any even-point metric.

Researchers have used these IRTree models to explore the influence of MRS and ERS on scale response data. Böckenholt (2017) found IRTrees modeled data of the Personal Need for Structure Scale (PNS) in both Likert and funnel response format. He noted the advantage of IRTrees is that the latent traits described in the models are more accurate than traditional IRT models that only consider trait measurements. Similarly, Böckenholt and Meiser (2017) also compared the IRTree framework to latent class modeling to explore response styles in the PNS. They found both methods detected response styles similarly. However, as the IRTree models do not assume ordinality, they therefore conclude the model is better at decomposing response styles from the latent trait being assessed.

## Current Study

In the current study, we explore the relationship of IER and response styles in the IRTree framework, both prior to and after cleaning the data. First, we explored the factor structure of IER with a variety of metrics to determine if two factors (random and non-random responding) comprise indirect IER with the metrics we have chosen for this study.

*H1: There are two factors that comprise indirect IER: random and non-random responding.*

Second, we explore the relationship of the latent variables from various IRTree models with random indirect, non-random indirect, and direct metrics of IER. We note an important issue arises when attempting to clean data for IER when exploring its relationship with response styles. Response styles are a typical way of responding, however the non-random metrics of IER, such as the long-string index, evaluate participants by non-variant response patterns. Given this and the association between ERS and IER (Grau et al., 2019), cleaning data based on non-random IER can unduly influence models. Similarly, cleaning data by random metrics may also unduly influence the results of the data as multivariate outliers may be actual responses to the scales provided. As such, although we assess direct, indirect random, and indirect non-random IER in our sample, we only relied on direct metrics of IER for cleaning, as they are clear indicators of insufficient effort or attention that are not potentially contaminated by response styles. We expected that this cleaning process would not only improve model fit for our IRTree analyses, but also elucidate how the relationship between IER and RS might change as a result of data cleaning.

For the IRTree analyses, we sought to examine a variety of scale types and factor structures. To that end, we largely turned to preexisting IRTrees research. First, we considered the Personal Need for Structure Scale (PNS; Thompson et al., 2001), due to the APP model being based on a 6-point scale and previous research having used that exact scale to investigate the model (Böckenholt, 2017). Similarly, we examined the General Self-Efficacy Scale (GSE; Schwarzer and Jerusalem, 1995) based on the literature proposing the use of the APP with 4-point scales (Meiser et al., 2019). Böckenholt (2019) had previously applied the MPP model to the shortened Need for Cognition Scale (NFC; Cacioppo et al., 1984), which also carried the distinct features of using a 5-point scale and half of the items being reverse-coded. Finally, to incorporate single-factor, non-reversed coded scales into our analyses, we administered both scales from the Positive and Negative Affect Schedule (PANAS; Watson et al., 1988). By examining multiple IRTree models across scales that vary substantially in content, we hoped that we would not only be able to examine response styles as they manifest across scales, but also hope to identify trends or patterns that emerged between response styles and IER metrics across IRTree model and scale type.

*H2: There is a positive relationship between IER and response styles.*

Given that the process of data cleaning inherently involves the removal of cases that exhibit IER in at least one form or another, it is unclear how that process might affect its relationship with response styles within the remaining cleaned data. It may be that standard IER cleaning metrics do not select out response styles in data in the same manner or degree as IER, which may in turn influence the relationship it has with IER once the data has been cleaned. As we did not have any theoretical basis for whether data cleaning may impact this relationship or not, we posed our final point of investigation as a research question.

*Research Question 1: Does that relationship the relationship between IER and response styles change following data cleaning?*

## Materials and Methods

### Participants

Research participants were recruited from Amazon’s Mechanical Turk (MTurk) platform. We used the CloudResearch (Litman et al., 2016) platform to collect data from MTurk as it offers an array of participant screening and approval options. The study involved the completion of an online survey battery, which took approximately 20 min to finish. All participants who submitted a completed survey received $3.00 as compensation. Because we examined participant data before and after data cleaning, all participants were compensated for completing the study, regardless of whether their data were removed following the cleaning procedure. All participants were required to be at least 18 years old, live in the United States, and be proficient in the English language. A total of 743 participants finished and submitted their responses for this study. Of the initial sample, 62.0% were male and the mean age was 36.6 years (*SD* = 9.8). Approximately 64.9% were Caucasian, 17.4% were black or African-American, 7.9% were Hispanic or Latino, 4.9% were Native American, 2.9% were Asian-American or Pacific Islander, and 1.8% identified as an ethnicity not listed. Following data cleaning using the direct IER criterion, which we describe below, 336 participants were removed from the sample leaving 407 participants in the cleaned sample. In this cleaned sample, 60.9% were male and the mean age was 36.9 (*SD* = 9.9). Approximately 72.2% were Caucasian, 11.3% were black or African–American, 5.2% were Hispanic or Latino, 5.2% were Native American, 4.7% were Asian–American or Pacific Islander, and 1.5% identified as an ethnicity not listed. We note the large difference in the cleaned data sample is because of accepting all participants that applied to take the study, which led to jettisoning over half the participants due to careless responding. Although typically this would be disparaged, the focus of the study was on IER. This indicates that we had sufficient IER to perform our analyses.

### Materials

#### Demographics

All participants were asked about their gender, age, ethnicity, and their proficiency in the English language.

#### Personal Need for Structure Scale

The Personal Need for Structure Scale (PNS; Thompson et al., 2001) is a 12-item survey (with four reverse-coded items) on a 6-point Likert scale ranging from one (*Strongly Disagree*) to six (*Strongly Agree*), which assesses the participant’s preference for predictability, consistency, and structure. The scale is comprised of two factors which include desire for structure and response to lack of structure (Svecova and Pavlovicova, 2016).

#### General Self-Efficacy Scales

The General Self-Efficacy Scales (GSE; Schwarzer and Jerusalem, 1995) is a 10-item unidimensional scale that assesses the participant’s overall confidence in their problem-solving abilities, coping skills, and resourcefulness. The scale is on a 4-point Likert scale ranging from one (*Not at all true*) to four (*Exactly true*).

#### Positive and Negative Affect Schedule

The Positive and Negative Affect Schedule (PANAS; Watson et al., 1988) is a 20-item scale that assesses positive (PA) and negative affect (NA) through asking the participant to what degree they have experienced a range of specific emotions and feelings that day, using a 5-point Likert scale ranging from one (*Very Slightly or Not at All*) to five (*Extremely*). Due to the orthogonal factor structure of PA and NA, we treated them each as separate scales for our analyses.

#### Need for Cognition Scale – Short Form

The Need for Cognition Scale – Short Form (NFC; Cacioppo et al., 1984) is an 18-item unidimensional scale (with nine reversed-scored items) on a 5-point Likert scale ranging from one (*Strongly Disagree*) to five (*Strongly Agree*), which assesses the participant’s general preference for thinking, engaging in intellectual activity, and being challenged cognitively.

#### Insufficient Effort Responding Battery

Although the scales described above were the referent scales for the analyses, inclusion of only those scales would limit our ability to effectively calculate all IER metrics of interest. As such, multiple surveys were combined and incorporated into a larger block of items to better assess participant IER metrics. All surveys in this block used a 5-point Likert scale ranging from one (*Strongly Disagree*) to five (*Strongly Agree*). This included the Locus of Control with Automation Scale (LoCA; Edwards, 2019), the Perfect Automation Schema Scale (PAS; Merritt et al., 2015), the Updated Perfect Automation Schema Scale (uPAS; [author blinded]), the Robot-Liking Scale (Katz and Halpern, 2014), and the Dispositional Trust Scale (Goldberg, 1992). The NFC was also integrated into this block. Apart from the initial items for LoCA, which includes a brief definition of automation and selection options for types of automation the participants use regularly, all items within these scales were randomized. This randomized order was kept consistent for all participants and presented across four pages in 19- to 20-item blocks.

### Insufficient Effort Responding Metrics

#### Direct Measures

We assessed IER directly with page time criterion (Huang et al., 2012), an Infrequency Scale (Huang et al., 2015), and survey instruction items (Ward and Pond, 2015). For each page of the survey, we measured completion time to determine whether participants were spending sufficient time to thoughtfully respond to each item. We used the criteria of less than 2 s per item, on average, to identify IER, as established in previous research (Huang et al., 2012). A total of eight survey pages were used for this metric. Additionally, we incorporated an 8-item Infrequency Scale (Huang et al., 2015) into the larger IER battery. The Infrequency Scale is an eight-item measure designed to assess IER by containing statements that are absurd, impossible, or would otherwise be extremely unlikely to be endorsed by an attentive respondent. An example item is “I can teleport across time and space.” We also incorporated three Survey Instruction Items into the larger IER battery, per Ward and Pond (2015). Survey Instruction Items are items that directly ask the participant to provide a specific response, thereby identifying IER whenever a participant fails to provide the correct answer. An example item is “For this item, please select ‘Strongly Agree’.” Page time and survey response flags (19 in total) were then summated to generate a direct IER variable, as well as used when applying a cutoff value for data cleaning, as described below.

#### Indirect Measures

As mentioned earlier, we hypothesized two categories of indirect metrics for assessing IER, random and non-random responding. To assess random responding we used the even-odd consistency index, Mahalanobis D, the psychometric synonym index, as well as the person-fit indices of standardized log-likelihood and Guttman errors (see Supplementary Material 1 for descriptions of each metric). To assess non-random responding we used the long-string index and the average long-string index. Metrics were generated using functions from the *careless* (Yentes and Wilhelm, 2021) and *PerFit* (Tendeiro et al., 2016) packages for R and RStudio. All indirect metrics and indices were derived from the survey response data (excluding demographics) of each sample prior to cleaning. No indirect measures of IER were used in the data cleaning process.

#### Random Responding Measures

We used even-odd consistency (Meade and Craig, 2012), Mahalanobis D (Mahalanobis, 1936), psychometric synonyms (Meade and Craig, 2012), the standardized log-likelihood index (*l*_*z*_; Karabatsos, 2003), and the Guttman Error index (*G*; Emons, 2008) to assess random responding.

#### Non-random Responding Measures

Long-string indices are commonly employed to identify participants who provide the same response to an extended series of items, regardless of their content (Johnson, 2005). Long-string values are calculated by taking the responses for each item in sequence, then determining the longest length of repeated responses for each page. The average long-string scores for each page are used to create an average long-string index. The maximum long-string index is an alternative method of identifying non-random IER (Meade and Craig, 2012), determined by identifying the greatest long-string length across all pages of the survey.

### Data Cleaning

We used the flags created from the direct IER metrics for data cleaning. We took the eight page-time flags and marked all participants who were engaging in page-time IER on five or more pages (i.e., more than half of the survey pages). Likewise, we then created an overall survey response criterion for the 11 survey response flags by using the criterion of having five or more flagged responses across the Infrequency Scale items and survey response items. If participants received an overall flag for page time, survey response, or both, they were marked for cleaning and removed from the cleaned version of the data.

### Data Analysis

All analyses were conducted in R (Version 4.1.0) using RStudio (Version 1.4.1106). We used the *psych* package (Revelle, 2020) to conduct exploratory factor analyses and the *mirt* package (Chalmers, 2012) for IRT analyses. As there are no set criteria for IER metrics, we conducted exploratory factor analyses on the metrics to ensure we were measuring random and non-random IER. First, we calculated the primary and composite IER metrics for every participant. Next, we performed an exploratory factor analysis and composed a scree plot on the indirect IER metrics. Once the factors were established, we calculated the reliabilities for each and absolute fit statistics for the overall model.

Scale reliabilities were calculated for both versions of the data set. For all five major scales of interest (PNS, GSE, PA, NA, and NFC), we followed the same general sequence of analyses. First, we created IRTree models for each scale. To do so, we transformed the data using the methods described by De Boeck and Partchev (2012). This involved creating a new data matrix of pseudo items based on the decision nodes within the IRTree model of interest for each scale. A summary of the pseudo item matrices employed across the study can be found in Supplementary Material 2^1^. Following data transformation, the IRTree was then fitted with the 2PL model, allowing the response style factors to covary, and estimated with the MH-RM algorithm. We then extracted the factor scores of the IER EFA and the IRTree model, and correlated those scores with each other, participant demographics, and the overall direct IER variable. Finally, we took the correlations from the cleaned and uncleaned samples and compared them as independent groups to determine if correlations scores changed significantly following data cleaning.

## Results

### Insufficient Effort Responding Exploratory Factor Analysis

Using the uncleaned data, we conducted an EFA with oblimin rotation and maximum-likelihood estimation on the indirect IER metrics to determine their factor structure (see Table 1). We found mixed support for absolute model fit, with the model having acceptable values for chi-square (80.79), AIC (77.82), and BIC (40.94), but an RMSEA of 0.12. We used a cutoff value of 0.30 to determine whether a metric sufficiently loaded onto a factor. Results illustrated a two-factor solution fit well with random IER and non-random IER. Cronbach’s alpha for each of the factors were found to be acceptable (random IER α = 0.78; non-random IER α = 0.94). For the random IER metrics, all loaded onto a single factor except for even-odd consistency, which also negatively loaded onto the other factor, and psychometric synonyms, which did not load onto either factor. The average long-string and maximum long-string metrics loaded onto the other factor. However, the scree plot findings were ambiguous. Based on the slopes of the factor and component curves, the number of factors or components could be two or three (see Supplementary Material 3). However, only the first two factors and components had eigenvalues greater than 1, which led us to conclude that the two-factor solution was sufficient. The two-factor solution accounted for 72.0% of the initial variance and 67.0% of the extracted variance. The random IER factor had a significant negative correlation with non-random IER (β = –0.21, *p* < 0.05) but it was not significantly correlated with direct IER metrics (β = 0.05, *p* > 0.05). In contrast, the non-random IER factor demonstrated a significant negative correlation with the direct IER metrics (β = –0.23, *p* < 0.05). Hypothesis 1 was supported.

**TABLE 1:** Results of exploratory factor analysis for indirect IER metrics.

Indirect IER metric	λ_1_	λ_2_
Even-odd consistency	**0.314**	**–0.300**
Mahalanobis D	**0.902**	–0.038
Psychometric synonyms	0.079	–0.260
Standardized log-likelihood	**0.984**	–0.051
Guttman errors	**0.890**	0.114
Average long-string	–0.037	**0.883**
Maximum long-string	0.029	**1.003**

*N = 743. Factor loadings greater than | 0.3| are in bold.*

### Insufficient Effort Responding and Response Styles

#### Data With Insufficient Effort Responding Included

In order to explore Hypothesis 2, we explored the data across scales before cleaning for IER. The observed means, standard deviations, and internal consistency estimates for all scales prior to cleaning for IER and after cleaning for IER are illustrated in Table 2. All scales showed acceptable Cronbach’s alpha estimates both prior to and after cleaning for IER, and a paired-samples *t*-test comparing the cleaned and uncleaned reliabilities found they did not significantly change following cleaning, *t*(4) = –2.39, *p* = 0.075. Next, we conducted the structural analyses for each scale prior to and after cleaning for IER. Table 3 illustrates the Akaike information criterion (AIC), Bayesian information criterion (BIC), sample size adjusted BIC (SABIC), number of parameters estimated and the root mean square of error (RMSE) for each scale prior to cleaning. We note that there are no absolute fit indices available for IRTree models. Although we report the AIC, BIC, and SABIC they are not relevant to comparing models as they are not based on the same data (e.g., pre- and post-cleaning). However, recent researchers have advocated the RMSE for comparing models as it represents the error of the model in the data, regardless of differences between datasets (Ames and Myers, 2020). We note in Table 3, that almost all the RMSE values were lower post-cleaning, indicating less error associated with the model. We subsequently explored the correlation of the IER factors with the latent factors of the IRTrees models. Supplementary Materials 4–8 illustrate the full correlation matrices of the construct and IER metrics and descriptive statistics for each scale. For ease of interpretation, we have included all the correlations between the scale constructs and IER factors in Table 4.

**TABLE 2:** Descriptive statistics for scales of interest before and after cleaning.

	Before cleaning^1^			After cleaning^2^		
	*M*	*SD*	α	*M*	*SD*	α
PNS	4	0.68	0.78	4.04	0.84	0.87
GSE	3.1	0.46	0.83	3.13	0.49	0.88
PA	3.54	0.88	0.90	3.35	0.97	0.92
NA	2.44	1.22	0.96	1.74	0.89	0.95
NFC	3.18	0.63	0.87	3.31	0.77	0.92

*^1^N = 741–743.*

*^2^N = 406–407.*

*PNS, personal need for structure; GSE, general self-efficacy; PA, positive affect; NA, negative affect; NFC, need for cognition.*

**TABLE 3:** Fit statistics and parameters for IRTree models before and after cleaning.

		AIC		BIC		SABIC		RMSE	
Scale	Parameters	Uncleaned	Cleaned	Uncleaned	Cleaned	Uncleaned	Cleaned	Uncleaned	Cleaned
PNS^1,2^	94	22413.95	12250.62	22847.23	12627.22	22548.74	12328.94	1.06	1.02
GSE^3,4^	41	14179.89	6896.57	14368.93	7060.93	14238.74	6930.83	0.64	0.57
PA^1,4^	63	18639.21	10288.86	18929.60	10541.42	18729.55	10341.51	0.85	0.85
NA^1,4^	63	15218.96	6628.91	15509.35	6881.47	15309.30	6681.56	0.74	0.65
NFC^4,5^	111	32695.25	17476.28	33206.74	17921.26	32854.27	17569.04	0.92	0.91

*^1^Uncleaned n = 742.*

*^2^Cleaned n = 406.*

*^3^Uncleaned n = 743.*

*^4^Cleaned n = 407.*

*^5^Uncleaned n = 741.*

*PNS, personal need for structure; GSE, general self-efficacy; PA, positive affect; NA, negative affect; NFC, need for cognition.*

**TABLE 4:** Correlations between IER constructs and latent variables from IRTree models.

	Random IER			Non-random IER			Direct IER (Sum)		
Latent variables	Uncleaned	Cleaned	Diff. *p*	Uncleaned	Cleaned	Diff. *p*	Uncleaned	Cleaned	Diff. *p*
Agree W (PNS)	**–0.16**	0.02	**0.003**	–0.04	–0.04	0.999	**0.41**	–0.10	** > 0.001**
Agree S (PNS)	**–0.27**	–0.13	**0.018**	–0.09	–0.02	0.257	**0.44**	–0.11	** > 0.001**
Agree (GSE)	**–0.15**	–0.09	0.325	**0.24**	**0.27**	0.604	**–0.13**	**–0.30**	**0.004**
Agree (PA)	0.01	**0.17**	**0.009**	**–0.23**	**–0.30**	0.223	**0.37**	**0.19**	**0.002**
Agree (NA)	–0.02	0.10	0.051	**–0.27**	**–0.36**	0.106	**0.73**	**0.54**	** > 0.001**
Agree (NFC)	–0.06	**–0.23**	**0.005**	**0.16**	**0.18**	0.739	**–0.39**	**–0.27**	**0.029**
Midpoint (PNS)	**–0.27**	**–0.42**	**0.006**	–0.02	0.07	0.146	**–0.20**	0.04	** > 0.001**
Midpoint (PA)	**–0.22**	**–0.36**	**0.013**	–0.06	–0.05	0.871	–0.02	0.03	0.419
Midpoint (NA)	–0.07	0.00	0.257	**–0.27**	**–0.35**	0.152	**0.52**	**0.49**	0.515
Midpoint (NFC)	–0.08	–0.15	0.252	–0.10	–0.05	0.416	0.06	**0.26**	**0.001**
Extreme (PNS)	**0.42**	**0.55**	**0.006**	0.00	–0.10	0.105	**0.14**	–0.02	**0.009**
Extreme (GSE)	**0.27**	**0.29**	0.726	0.05	0.04	0.871	–0.04	–0.13	0.143
Extreme (PA)	**0.32**	**0.35**	0.585	**0.12**	**0.18**	0.321	–0.06	–0.09	0.626
Extreme (NA)	0.09	0.03	0.330	**0.26**	**0.34**	0.155	**–0.51**	**–0.48**	0.521
Extreme (NFC)	**0.49**	**0.52**	0.515	–0.04	–0.07	0.627	0.08	–0.09	**0.006**

*Significant correlations are in bold (p < 0.05 following Bonferroni alpha correction).*

*PNS, personal need for structure; GSE, general self-efficacy; PA, positive affect; NA, negative affect; NFC, need for cognition.*

*Diff. p column includes p-values for Fisher’s z correlation comparisons of uncleaned vs. cleaned samples (p < 0.05 are in bold).*

The random IER factor was negatively correlated with the agreement nodes for the PNS and GSE scales. Additionally, the random IER factor was negatively correlated with the midpoint node for the PNS and PA scales and positively related to the extreme node for all scales except for NA. The non-random IER factor demonstrated markedly different correlations. For the agreement node in the IRTrees models, the factor was positively correlated on the GSE and NFC, but negatively related with PA and NA. The non-random IER factor also negatively related to the midpoint node of NA and positively related to the extreme nodes of PA and NA. The direct IER variable was positively related to the agreement nodes on the PA, NA and PNS scales while negatively related to the same node for the GSE and NFC. Additionally, the direct IER variable was positively related to the NA midpoint and NFC extreme nodes, but negatively related to the PNS midpoint and NA extreme nodes. As the IER factors were positively related to only some of the response style nodes across scales, Hypothesis 2 was partially supported.

#### Data Cleaned for Direct Insufficient Effort Responding

Next, we examined the data after cleaning for IER to investigate Research Question 1. Table 3 illustrates the AIC, BIC, SABIC, number of parameters estimated and the RMSE for each scale after cleaning. Importantly, we note that we are unable to compare the model fit of each model before and after cleaning, as only relative fit indices are available for IRTrees. Furthermore, these indices are not comparable as they are modeled on different data (data with IER cases and data without). We considered the correlations of the IER constructs with the latent variables from the IRTree models in comparison to the pre-cleaning results, looking both at the significance of the correlations themselves and the difference between the two datasets. As illustrated in Table 4, the agreement nodes with random IER saw significant change for most latent constructs after cleaning. The correlation of random IER with the agreement nodes of PNS and GSE were no longer significant, however the difference in correlations was only significant for the PNS agreement nodes. Additionally, random IER had a significant positive relationship with PA agreement nodes and a negative relationship with the NFC agreement node, which were not significant prior to cleaning and were a statistically significant change. There was also a significant change to the correlation of the PNS midpoint, PNS extreme, and PA midpoint nodes with random IER, such that there was a stronger relationship after cleaning. In contrast, the non-random IER construct had similar correlations with the agreement, midpoint, and extreme nodes as it did prior to cleaning. Indeed, no significant differences were found for the non-random IER construct with any of the scale nodes.

Lastly, we report the correlations of the direct IER variable with the latent constructs extracted from the various models. However, we do not interpret these results for several reasons. First, because we cleaned the data based on a criterion within the direct IER variable, we essentially created a ceiling for this variable in the cleaned data, as any participants who met the cutoff criterion were removed. This eliminated a significant amount of variance within the scale itself. Second, as the construct only includes responses to individual items and page time, there is little theoretical justification for why it would be related to response styles beyond its association with indirect IER, which typically considers response patterns across the entire survey. Finally, because we did not incorporate it into the IER factor analysis (again due to its use as the cleaning criterion), we wish to exercise caution in interpreting its relationships within the broader nomological net of IER and response styles, as we do not have confirmation regarding its validity as a structure.

## Discussion

The accurate measurement of psychological constructs is imperative to research in not only psychology, but a variety of fields, including management and information technology. The current paper explored the relationship of two confounding constructs of self-report data in the literature, IER and response styles. The current study is the first, to our knowledge, to explore the factor structure of IER with many of the most employed, relevant metrics and constructs in the literature. This enabled us to explore both random and non-random IER to determine the effect of each on responses styles, differentiating the causes of IER. The constructs of IER and response styles have similar underlying causes, mainly a lack of motivation or inability to respond to each item with full thoughtfulness and attentiveness. The current study found a modest correlation between IER and response styles, possibly because they have different underlying reasons for the lack of motivation. Whereas IER occurs when a respondent answers an item without reading an item, response styles are when a respondent responds to item superficially, engaging in a cognitive shortcut. It may be that the difference between the constructs is a matter of degree of cognitive effort while completing a survey. However, we were unable to explore the underlying causes for IER and responses styles in the current study.

### Insufficient Effort Responding Factor Structure

Research on IER has developed two general approaches to assessing survey responses: direct and indirect. Furthermore, the literature has delineated IER response patterns into two types, random and non-random responding, both of which are best observed through indirect methods. The current study employed a variety of metrics to assess IER, including both direct and indirect measures for both random and non-random responding. As we mentioned above, due to the difficulties in identifying the reason behind flagged direct IER responses and their relative lack of contamination with overall response patterns, we did not include them in our factor analyses. Our factor analyses illustrated two factors of indirect IER, random and non-random responding, confirming the theoretical outline by Curran (2016) and supporting Hypothesis 1. The random and non-random factors had differential relationships with the direct IER variable which remained stable after cleaning, further supporting the caution by Meade and Craig (2012) to include several types of IER metrics in a study to accurately identify IER in its various forms. However, it should be noted the non-random factor was primarily comprised of long-string indices, as both the long-string and average long-string are derived from the same IER assessment method. Exploration of additional types of non-random IER responding would greatly help the understanding of IER.

Although we found two factors for indirect IER, their exact relationship with direct IER was beyond the scope of the current study. Direct metrics can detect who is performing IER, but indirect metrics explain how and can infer why. Despite this, the correlations (or lack thereof) between the direct IER metrics and indirect IER indices remained stable before and after cleaning. In the case of non-random IER, which had a significant negative relationship with direct IER, this could be attributable to the tendency to completely ignore direct IER item prompts in favor of maintaining a repeated response. In contrast, there was no significant correlation with the direct IER metrics and random IER index. It could be that those who engage in random IER patterns may be able to correctly answer some direct IER questions by chance, while missing other such items entirely. True to their overall tendency to respond randomly across the scale, random IER responders may also respond randomly to direct IER items, eliminating any apparent correlation within that portion of the battery. It may also be that participants who are “survey-savvy” may respond randomly throughout the survey, while looking for attention checks. As they are expending little cognitive effort, they miss some of the attention checks, leading to the lack of relationship between the two metrics as the participant responds correctly to the attention checks sometimes, but other times may not. Additionally, if an attention check is engaged, the participant will have to read the item and cognitively process it to respond appropriately (e.g., responding with a response of “4”) which may also influence the page time metric.

### Random Insufficient Effort Responding and Response Styles

We found a relationship of IER with response styles in the current study, supporting Hypothesis 2, although the relationship may have been occasionally spurious. The current study included several random IER metrics to comprise a factor of random IER. We found the random IER factor was negatively related to MRS for several scales. This is in line with previous investigations of MRS and IER, which found similar correlations (Grau et al., 2019; Conijn et al., 2020). The negative relationship between the random IER and MRS found in this study may be a function of the lack of responses options of MRS and random IER. In other words, there is only one option for the MRS variable on an odd-point scale, such as the commonly employed 5- or 7-point scales, whereas there are two options for ERS (e.g., response option “1” and “5” in a 5-point Likert scale). For odd-point scales (which comprised most items in this study), a respondent consistently choosing the midpoint is conceptually closer to non-random responding, as well as being more likely to be interpreted as non-random responding by the currently employed metrics. It should be noted that the literature is not entirely consistent, as this contrasts with other research that did not find a significant association of overall IER and MRS (Grau et al., 2019). This study comprised several metrics of direct, indirect random responding, and indirect non-random IER responding. As such, the different relationships of random and non-random responding may have influenced the overall IER correlation and MRS.

Random IER was also positively related to ERS, which is comparable to previous research on IER and ERS (Grau et al., 2019; Conijn et al., 2020). Like ERS, random IER is not bounded by directionality. In other words, as how ERS allows for responses on opposite ends of the scale, random IER patterns allow for varied responses across the entire length of the scale. Respondents wishing to exert little cognitive effort in responding while still maintaining a superficial appearance of effort may employ a response style. Therefore, a respondent who tends to engage in random IER may prefer the ERS over other response styles, as it allows for responses in both directions for a bidirectional scale. In such cases, having responses on opposite ends of the scale creates an appearance of thoughtful responses but can be easily detected as a response style or IER using the metrics employed in this study.

Research has demonstrated respondents on online platforms may be more familiar with survey attention checks than some in-person samples, due to their elevated experience with survey-taking (Hauser and Schwarz, 2016). Such respondents may look for key words and phrases such as “not,” “no,” “please select,” or the affective connotation of the item as cues to respond, to help ensure they receive payment for the study. This may explain some of the overlap of the random IER and response styles. Due to being more “survey-savvy,” online responders may be able to escape detection by basic attention checks, while still only investing minimum effort and attention to complete the survey. Second, respondents that try to conserve resources by not evaluating the substance of the item may still respond with response styles to alleviate cognitive load and expedite the survey-taking process. In this sense, response styles may exist as a sort of compromise for individuals who do not want to exert full cognitive effort on a survey, while still providing responses that retain some substance that could be of value to the researcher. However, researchers should still account for the increased variance attributable to response styles, which may or may not be sufficient to influence their results (Baumgartner and Steenkamp, 2001).

In general, random IER had a spurious relationship with the trait of interest being measured, that is, the construct the scale was created to assess. In the IRTree models, random IER was negatively related to the constructs of PNS and GSE prior to cleaning. Interestingly, these are the only two even-point scales. It may be that even-point Likert scales are more amenable to random IER due to the lack of a true midpoint. Without a centered option, IER responders may be more inclined to engage in random over non-random IER. In contrast, random IER did not have a relationship with the latent construct extracted from the even-point scales prior to cleaning. The random responses may have influenced the item parameters (i.e., discrimination and slopes) but not the overall estimation of theta for the model. Random IER adds random variance to the estimation of the item parameters but may not influence the overall theta extracted for the model. However, research has demonstrated random IER can influence person fit of the IRT model (Beck et al., 2019). Indeed, two of the metrics we used to determine random IER are person fit statistics, the standardized log likelihood (*l*_*z*_) and Guttman errors. Interestingly, *l*_*z*_ incorporates theta into its estimation, though the factor loading of *l*_*z*_ into the random factor may have been small enough that theta’s relationship with random IER was negligible. Taken together, these results suggest that random IER patterns are more negatively related to MRS, more positively related to ERS, and potentially create spurious relationships with the scale construct itself. When considering response styles or IER, researchers should give closer evaluation to instances of high ERS, as they may also be engaging in random IER.

### Non-random Insufficient Effort Responding and Response Styles

The non-random IER metric used in the current study, and in IER research in general, is based on the long-string index, a measure of straight-lining on a scale. Overall, the non-random IER metric was not related to MRS, which contrasts with what we would expect from a theoretical perspective. Participants that tend to respond with a midpoint should have a higher long-string index as they are repeatedly choosing the midpoint. However, the only significant effects for MRS with non-random IER were a negative relationship with negative affect and need for cognition, though the significant correlation vanished for need for cognition after cleaning. It is possible the lack of correlation between MRS and non-random IER could be attributable to scale structure for the PNS, as the midpoint node includes multiple scale points in the IRTree model for PNS, its possible for someone to employ a MRS on that scale without long-string responding. However, this does not account for lack of correlations across the five-point scales, excluding negative affect. Regardless, it should be noted the lack of relationship for non-random IER and MRS aligns with previous research (Grau et al., 2019).

Non-random IER was positively related to ERS in the Positive and Negative Affect scales before and after cleaning, but no other relationships were significant. Again, this is similar to results found by Grau et al. (2019), who found no significant relationship between the long-string index and ERS. However, non-random IER had significant relationships with the latent traits of every scale except the PNS scale prior to cleaning. As noted by Kam and Meyer (2015), IER can interact with the type of survey. IER can combine with the latent trait being assessed to distort factor analyses. Additionally, the appearance of non-random IER (in contrast to other forms of IER) may be driven by the qualities of the underlying construct itself. Most of the constructs assessed in the current study were constructs that are socially desirable. This may have led participants to prefer a non-random IER pattern of agreement with the latent construct, which may be associated with acquiescence response style (see Van Vaerenbergh and Thomas, 2013). Accordingly, researchers who are considering IER and response styles may wish to check for non-random IER independent of any response style analysis, due to the lack of relationship.

### Direct Insufficient Effort Responding and Response Styles

The direct IER metrics had inconsistent relationships with MRS, ERS and the latent trait being assessed. It is difficult to interpret the relationships of the direct IER metrics because the underlying cause of the IER is not known. As mentioned above, IER is theorized to be multifaceted (Kam and Meyer, 2015), having been correlated with myriad of respondent factors at both the state (Huang et al., 2015) and trait level (Bowling et al., 2016; DeSimone et al., 2020). This becomes especially difficult to decompose with direct IER patterns, as they are dependent on responses to singular items and time spent, rather than responses across the full breadth of the survey. Complicating this further, direct IER survey items are the only metric directly visible by the participants. In the case of online samples, experienced participants (the “survey-savvy,” as described by Hauser and Schwarz, 2016), may be trained in identifying these items and selecting the desired responses, despite engaging in minimal effort across the remainder of the survey. This would only serve to complicate IER detection on behalf of the researcher, as a participant may be capable of passing direct IER checks, while still responding carelessly by the standards of the indirect metrics.

The spuriousness of the relationships and complexities unique to each form of IER illustrates another reason for Meade and Craig’s (2012) advice to utilize both indirect and direct measures, which is an understanding of the type of IER occurring for individuals or across the scales. It remains to be seen if a person can perform both random and non-random IER simultaneously or across a single survey, and if so, what that would imply for the constructs of IER and how we assess them. Additionally, certain scales, such as those that measure socially desirable traits, may facilitate non-random IER whereas other scales that do not necessarily have a socially desirable aspect, may facilitate random IER. It may be that participants read the first few items and construct a schema on how to respond to the items but engage in less effort as they progress through the survey, regardless of pattern (Bowling et al., 2020). Ultimately, while it may be that direct IER is the least-related form of IER to response styles, it can still be assessed within a larger battery of IER assessment, contributing to the holistic determination of IER for participants.

### Relationships Post-cleaning

Interestingly, most of the IER-response style relationships were still significant after cleaning the data. Indeed, the random and non-random IER factors maintained all significant relationships with the MRS and ERS factors they had prior to cleaning. Likewise, the direct IER construct also retained most relationships with the MRS and ERS factors it had prior to cleaning. Lastly, most of the IER metrics relationship with the agreement factors (i.e., trait of interest) remained, as well. The one exception to this was random IER, which when was cleaned, the random IER no longer was associated with the agreement factors for PNS or GSE. Although it should be noted after cleaning there were two new relationships of random IER with the PA and NFC agreement factors.

There are several possible reasons why only random IER demonstrated a change in relationships post-cleaning. It could be that there is a deep relationship with response styles and the IER metrics that remains after cleaning has occurred, at least for non-random and direct IER. This may indicate these IER metrics and response styles may share an underlying similar process, or they may be similar in detection methods. Alternatively, it could be that those who were cleaned out were primarily random IER employers, while more “survey-savvy” participants (Hauser and Schwarz, 2016) engaged in non-random IER patterns. As such, participants who employ random IER may not even give enough attention and effort to avoid incorrect responses on attention checks, leading to them being more likely to be cleaned out and subsequently affecting the random IER correlations post-cleaning. Regardless, these results call into question the utility of relying solely on attention checks when cleaning data for IER from a practical standpoint. Given the limited change in relationships between the IER constructs and RS factors, this would suggest that a substantial portion of the sample remained that engaged in indirect IER to the degree that the quality of the data may have been impacted, even after removing individuals who had failed the attention check items. Once again, this reinforces the importance of employing multiple IER metrics when cleaning, as relying on individual metrics or types of check alone may not be sufficient to ensure high-quality data (Curran, 2016).

### Limitations and Future Directions

The current study has several limitations. First, we chose to clean the data by employing direct IER metrics, despite recommendations in the literature to employ varied methods with data cleaning (e.g., Meade and Craig, 2012; Curran, 2016). We deliberately chose this cleaning method due to our interest in IER’s relationship with response styles and IRTrees. Unlike the various indirect IER metrics, direct IER metrics do not assess response patterns across scales and surveys overall but rather are based on responses to individual items distributed throughout a survey or on metrics secondary to response patterns. While our intention was that this would sufficiently remove IER cases from the data without inordinately biasing the response style and IRTree results of the cleaned data set, it is inevitable that some cases would be missed through cleaning with direct metrics alone. This limitation is mitigated by our efforts to employ direct metrics from multiple scales and types (item responses and page time). Future research interested in the relationships between IER, response styles, and IRT should continue to investigate methods of detecting IER that are not potentially contaminated by specific response patterns.

Second, given the literature supporting IER’s connection to stable traits (e.g., Bowling et al., 2016; DeSimone et al., 2020), data cleaning via IER detection metrics without potentially removing a unique subset from the sample is a challenge that the literature has yet to fully address. Research investigating IRTree modeling’s relationship to personality traits is currently scant (e.g., Ames and Myers, 2020), our results suggest that by way of IER and response style’s relationship with IRTrees, IER personality may indirectly influence said models. Future research in IER and response styles should continue to investigate these behavioral patterns as a reflection of underlying respondent traits and likewise seek out data cleaning and IER detection methods that are minimally influenced by respondent traits.

Third, the current study lacked a standardized battery for IER validation. It has been repeatedly emphasized throughout the IER literature that selection of IER metrics should be considered carefully, with metrics chosen based on the unique qualities of their survey and experimental design (Meade and Craig, 2012; Huang et al., 2015; Curran, 2016). However, aside from general guidelines based on the estimation parameters of each individual metric, there is no standard criteria for IER detection. While we sought to cover a range of metrics of varying types and categories, our chosen metrics were non-exhaustive of the IER detection techniques available. Furthermore, while our IER results supported previous literature distinguishing the factor structure of different IER types (Meade and Craig, 2012), there are currently limitations across the literature for detection methods, particularly with detection of non-random IER. As previously stated, current methods of non-random IER detection are almost exclusively variations of the long-string index. However, we propose that response styles and other forms of repeated, patterned responses may also be considered as an alternative to non-random IER detection. Recent research into the phenomenon of “anchoring” (choosing an item response based on its proximity to the response of a previous item) suggests there may more robust methods of examining non-random IER and response styles than what is most typically employed (Lyu and Bolt, 2021). While it was beyond the scope of the current paper, future research in IER will wish to investigate and validate these alternative methods of non-random IER methods, as well as continue to work toward standardized criteria and test batteries of IER detection to enhance data cleaning techniques across all domains of survey and experimental research.

Lastly, the current study focused on IRTrees to model response styles. However, it should be acknowledged that other methods of assessing response style exist in the literature. Models such as the modified generalized partial credit model (mPCM; Jin and Wang, 2014) and multidimensional nominal response model (MNRM; Johnson and Bolt, 2010; Wetzel and Carstensen, 2017) also assess response styles from an IRT perspective. All three of the perspectives use latent traits to assess response styles, though the models themselves vary. Future research will want to replicate these findings with other methodologies to more accurately understand the relationship of IER with response styles. Indeed, measuring response styles has proven problematic in the literature with many different measures and methods of assessing the constructs. Recent literature has begun to compare the methods more directly, in one case finding that both the MNRM and IRTree methodologies are comparably effective at measuring response styles, with the mPCM not being appropriate under some circumstances (Zhang and Wang, 2020). Regardless, additional research is needed to continue validating these methods, as well as applying them to related domains of investigation, such as IER.

### Implications

There are several implications of the current study for both IER and response styles. First, the direct IER, random indirect IER, and non-random indirect IER metrics all demonstrated differential relationships, suggesting they are separate constructs that must be independently accounted for during data cleaning. It remains to be seen what the underlying process is of the direct IER metric, as it surprisingly did not have a relationship with the random indirect IER metric. Indeed, a majority of the indirect IER relationships were still significant after cleaning for direct IER. As such, we reiterate Meade and Craig’s (2012) suggestion of using varying types of IER checks. Specifically, we advocate metrics that encompass all three constructs. Second, we established a relationship of IER and response styles. Both are theorized to be methods for conserving cognitive effort, indicating there may be overlap of the underlying psychological processes that lead to the constructs. We found modest relationships among the response style variables and IER constructs, but not so strong as to indicate the same process. Furthermore, we found a majority of the IER constructs were still related to the response style constructs after cleaning. Again, this may be because IER and response styles are driven by similar underlying cognitive processes, namely conserving resources. For that same reason, it may also be the indices used to detect indirect IER are sensitive to response styles. While we were unable to elucidate the full impact of IER metrics on detecting and removing response styles in this study, the current research affirms the interconnectedness of these constructs.

## Conclusion

Response style and IER demonstrated a significant relationship and are both capable of influencing one another. Based on this, there may be some evidence to support the notion that participants who engage in IER and participants who employ response styles are both completing surveys across a spectrum of reduced effort, but further investigation is required. Both IER and response styles remain problematic nuisance variables for self-report studies for all fields that employ them, but this study represents an initial investigation into their association and shared influence, particularly within the nascent modeling framework of IRTrees. For that growing body of literature, this study emphasizes the importance of considering IER within our analyses while applying the framework, considering the relationship IRTrees carries with IER and how it can influence results. As we continue to advance our understanding of self-report survey data and develop new methods of analysis and modeling, it remains crucial that we continue to examine how variables such as IER and response styles may affect our findings and potentially lead us astray.

## Data Availability Statement

The original contributions presented in the study are included in the article/Supplementary Material, further inquiries can be directed to the corresponding author/s.

## Ethics Statement

The studies involving human participants were reviewed and approved by AFRL Institutional Review Board. The patients/participants provided their written informed consent to participate in this study.

## Author Contributions

GA designed the study, analyzed the results, and wrote the manuscript. ML analyzed the results and wrote the manuscript. Both authors contributed to the article and approved the submitted version.

## Conflict of Interest

ML is employed by General Dynamics Information Technology. This study received funding from the United States Air Force. GA is a research psychologist for the United States Air Force, which funded the project. The funder had the following involvement with the study: study design, implementation, data collection, analysis, and write up.

## Publisher’s Note

All claims expressed in this article are solely those of the authors and do not necessarily represent those of their affiliated organizations, or those of the publisher, the editors and the reviewers. Any product that may be evaluated in this article, or claim that may be made by its manufacturer, is not guaranteed or endorsed by the publisher.

## Footnotes

1 Researchers interested in the full data transformation and analysis process for IRTrees may refer to the R Markdown file included in the Supplementary Material for this article.
