# Supplementary material for: The Relationship of Insufficient Effort Responding and Response Styles: An Online Experiment
Source: Front Psychol. 2022 Jan 12;12:784375. doi: 10.3389/fpsyg.2021.784375 (PMC8789874; doi:10.3389/fpsyg.2021.784375)
Supplement: Supplementary file 1 [file Data-Sheet_1.docx]

| Indirect Metric | Description | References |
| --- | --- | --- |
| Even-Odd Consistency | All scales/scale factors are divided by their even and odd-numbered items, then the correlation between the even and odd items are calculated. Lower scores indicate poorer responding. | Jackson, 1976, 1977  Johnson, 2005  Meade & Craig, 2012  Huang et al., 2014 |
| Mahalanobis Distance | Method of outlier detection which determines a person’s distance from the center of a multivariate distribution. Higher values indicate greater outlying. | Mahalanobis, 1936  Ehlers et al., 2009  Maesschalck et al., 2009 |
| Psychometric Synonyms | The most highly correlated item pairs (r > .60 or 30 pairs is recommended) across the sample are identified and the within-person correlation for these pairs is calculated. Lower values indicate poorer responding. | Johnson, 2005  Meade & Craig, 2012 |
| Standardized Log-Likelihood | An IRT-based person-fit statistic which measures the likelihood of a person’s response pattern relative to the expected response pattern. Lower values are considered to indicate low-quality responding. | Drasgow, Levine, and Williams, 1985  Reise & Widaman, 1999  Karabatsos, 2003 |
| Guttman Errors | An IRT-based person-fit statistic which items are sorted by difficulty. Instances in which more difficult items are answered correctly while easier items are not are considered errors. Higher values indicate poorer responding. For ease of interpretation, raw error scores were standardized. | Guttman, 1944; 1950  Molenaar, 1991  Karabatsos, 2003  Emons, 2008 |
| Average Long-String | Maximum number of consecutive item responses per survey page are counted and then averaged across all pages. Higher scores indicate more repetitive responding. | Johnson, 2005  Costa & McCrae, 2008  Curran et al., 2010  Meade & Craig, 2012 |
| Maximum Long-String | Maximum number of consecutive item responses across the entire survey. Higher scores indicate more repetitive responding. | Johnson, 2005  Costa & McCrae, 2008  Curran et al., 2010  Meade & Craig, 2012 |

1. Pseudo-Items for Five-Category Model.

| Category | *Midpoint* | *Agreement* | *Extreme* |
| --- | --- | --- | --- |
| 1 | 0 | 0 | 1 |
| 2 | 0 | 0 | 0 |
| 3 | 1 | – | – |
| 4 | 0 | 1 | 0 |
| 5 | 0 | 1 | 1 |

2. Pseudo-Items for Six- Category Model.

| Category | *Midpoint* | *Weak Agreement* | *Strong Agreement* | *Extreme* |
| --- | --- | --- | --- | --- |
| 1 | 0 | – | 0 | 1 |
| 2 | 0 | – | 0 | 0 |
| 3 | 1 | 0 | – | – |
| 4 | 1 | 1 | – | – |
| 5 | 0 | – | 1 | 0 |
| 6 | 0 | – | 1 | 1 |

3. Pseudo-Items for Four- Category Model.

| Category | *Agreement* | *Extreme* |
| --- | --- | --- |
| 1 | 0 | 1 |
| 2 | 0 | 0 |
| 3 | 1 | 0 |
| 4 | 1 | 1 |


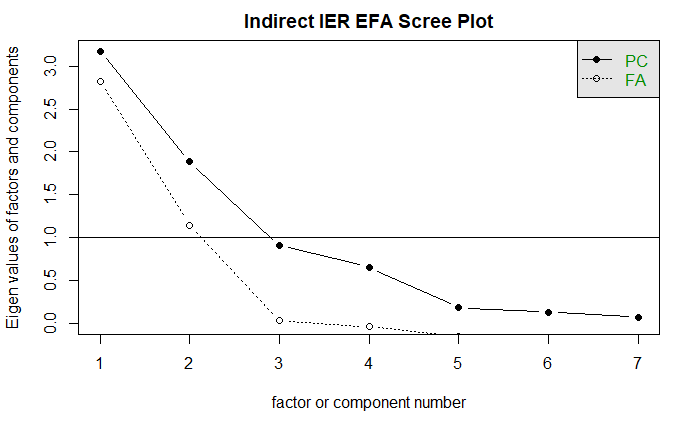


*Note.* Eigen values were calculated and plotted using the “scree” function from the *psych* package (Revelle, 2020).

|  | 1. | 2. | 3. | 4. | 5. | 6. | 7. | 8. | 9. | 10. | 11. | 12. | 13. |
| --- | --- | --- | --- | --- | --- | --- | --- | --- | --- | --- | --- | --- | --- |
| 1. Gender | – | **-.22** | .02 | -.12 | -.05 | -.11 | .06 | -.01 | -.02 | .03 | -.11 | -.13 | .00 |
| 2. Age | **-.14** | – | .04 | .05 | .08 | .06 | -.04 | -.08 | -.11 | -.08 | .05 | .05 | .07 |
| 3. English Proficiency | .05 | .02 | – | .06 | .09 | .09 | -.07 | .10 | **-.21** | -.03 | .08 | .09 | .01 |
| 4. PNS (RLS) | -.06 | .05 | .07 | – | **.59** | **.95** | .02 | -.07 | -.10 | **-.46** | **.89** | **.89** | **.36** |
| 5. PNS (DFS) | .01 | .08 | .07 | **.53** | – | **.79** | .10 | -.03 | -.14 | **-.49** | **.66** | **.63** | **.45** |
| 6. PNS (Total) | -.05 | .06 | .08 | **.94** | **.76** | – | .04 | -.03 | -.15 | **-.52** | **.90** | **.89** | **.43** |
| 7. Random IER | .04 | -.03 | -.04 | -.04 | .01 | -.01 | – | **-.21** | .13 | **-.42** | .02 | -.13 | **.55** |
| 8. Non-Random IER | -.03 | -.08 | .06 | -.03 | .04 | .02 | **-.21** | – | **-.30** | .07 | -.04 | -.02 | -.10 |
| 9. Direct IER Total | .03 | -.03 | **-.23** | -.01 | -.09 | -.10 | .05 | **-.23** | – | .04 | -.10 | -.11 | -.02 |
| 10. IRTree Midpoint | -.07 | -.09 | -.02 | **-.40** | **-.48** | **-.46** | **-.27** | -.02 | **-.20** | – | **-.54** | **-.33** | **-.92** |
| 11. IRTree Agreement (Weak) | .01 | .04 | -.03 | **.63** | **.44** | **.58** | **-.16** | -.04 | **.41** | **-.60** | – | **.96** | **.42** |
| 12. IRTree Agreement (Strong) | -.03 | .02 | -.05 | **.61** | **.35** | **.52** | **-.27** | -.09 | **.44** | **-.31** | **.93** | – | **.20** |
| 13. IRTree Extreme | .07 | .08 | .02 | **.34** | **.47** | **.41** | **.42** | .00 | **.14** | **-.94** | **.44** | **.18** | – |

*Note.* Correlations for data prior to cleaning are below the diagonal, correlations after cleaning are above the diagonal. Uncleaned *n* = 741-742; cleaned *n* = 405-406. Significant correlations are in bold (*p* < .05 following Bonferroni alpha correction).

|  | 1. | 2. | 3. | 4. | 5. | 6. | 7. | 8. | 9. |
| --- | --- | --- | --- | --- | --- | --- | --- | --- | --- |
| 1. Gender | – | **-.22** | .03 | .03 | .06 | -.01 | -.02 | .01 | .02 |
| 2. Age | **-.14** | – | .04 | .15 | -.04 | -.07 | -.11 | .16 | .11 |
| 3. English Proficiency | .05 | .02 | – | .11 | -.07 | .10 | **-.21** | **.17** | .04 |
| 4. GSE | .08 | **.15** | .07 | – | .03 | .13 | **-.20** | **.93** | **.81** |
| 5. Random IER | .04 | -.03 | -.04 | -.05 | – | **-.21** | .13 | -.09 | **.29** |
| 6. Non-Random IER | -.03 | -.08 | .06 | .12 | **-.21** | – | **-.30** | **.27** | .04 |
| 7. Direct IER Total | .03 | -.03 | **-.23** | -.04 | .05 | **-.23** | – | **-.30** | -.13 |
| 8. IRTree Agreement | .07 | **.14** | .11 | **.94** | **-.15** | **.24** | **-.13** | – | **.70** |
| 9. IRTree Extreme | .08 | **.**11 | .07 | **.80** | **.27** | .05 | -.04 | **.74** | – |

*Note.* Correlations for data prior to cleaning are below the diagonal, correlations after cleaning are above the diagonal. Uncleaned *n* = 742-743; cleaned *n* = 406-407. Significant correlations are in bold (*p* < .05 following Bonferroni alpha correction).

|  | 1. | 2. | 3. | 4. | 5. | 6. | 7. | 8. | 9. | 10. |
| --- | --- | --- | --- | --- | --- | --- | --- | --- | --- | --- |
| 1. Gender | – | **-.22** | .03 | .07 | .06 | -.01 | -.02 | -.03 | .06 | .03 |
| 2. Age | **-.14** | – | .04 | .12 | -.04 | -.07 | -.11 | .06 | .08 | .00 |
| 3. English Proficiency | .04 | .02 | – | -.11 | -.07 | .10 | **-.21** | .04 | -.09 | -.01 |
| 4. PA | .09 | .12 | -.05 | – | **.19** | **-.32** | .13 | **-.52** | **.94** | **.18** |
| 5. Random IER | .03 | -.03 | -.04 | .10 | – | **-.21** | .13 | **-.36** | **.17** | **.35** |
| 6. Non-Random IER | -.03 | -.08 | .06 | **-.23** | **-.21** | – | **-.30** | -.05 | **-.30** | **.18** |
| 7. Direct IER Total | .03 | -.03 | **-.23** | **.32** | .05 | **-.23** | – | .03 | **.19** | -.09 |
| 8. IRTree Midpoint | -.06 | -.09 | -.06 | **-.51** | **-.22** | -.06 | -.02 | – | **-.48** | **-.87** |
| 9. IRTree Agreement | .09 | .08 | -.05 | **.93** | .01 | **-.23** | **.37** | **-.45** | – | .08 |
| 10. IRTree Extreme | .07 | .05 | .08 | **.25** | **.32** | **.12** | -.06 | **-.86** | **.13** | – |

*Note.* Correlations for data prior to cleaning are below the diagonal, correlations after cleaning are above the diagonal. Uncleaned *n* = 741-742; cleaned *n* = 406-407. Significant correlations are in bold (*p* < .05 following Bonferroni alpha correction).

|  | 1. | 2. | 3. | 4. | 5. | 6. | 7. | 8. | 9. | 10. |
| --- | --- | --- | --- | --- | --- | --- | --- | --- | --- | --- |
| 1. Gender | – | **-.22** | .03 | -.04 | .06 | -.01 | -.02 | -.03 | -.02 | .02 |
| 2. Age | **-.14** | – | .04 | -.07 | -.04 | -.07 | -.11 | -.11 | -.09 | .11 |
| 3. English Proficiency | .04 | .02 | – | **-.28** | -.07 | .10 | **-.21** | **-.24** | **-.24** | **.22** |
| 4. NA | .02 | -.04 | **-.23** | – | .11 | **-.35** | **.60** | **.86** | **.95** | **-.84** |
| 5. Random IER | .04 | -.03 | -.04 | .02 | – | **-.21** | .13 | .00 | .10 | .03 |
| 6. Non-Random IER | -.04 | -.08 | .06 | **-.31** | **-.21** | – | **-.30** | **-.35** | **-.36** | **.34** |
| 7. Direct IER Total | .03 | -.03 | **-.23** | **.75** | .05 | **-.23** | – | **.49** | **.54** | **-.48** |
| 8. IRTree Midpoint | -.05 | -.08 | **-.23** | **.72** | -.07 | **-.27** | **.52** | – | **.94** | **-.99** |
| 9. IRTree Agreement | .01 | -.05 | **-.23** | **.95** | -.02 | **-.27** | **.73** | **.82** | – | **-.93** |
| 10. IRTree Extreme | .04 | .08 | **.21** | **-.71** | .09 | **.26** | **-.51** | **-.99** | **-.81** | – |

*Note.* Correlations for data prior to cleaning are below the diagonal, correlations after cleaning are above the diagonal. Uncleaned *n* = 741-742; cleaned *n* = 406-407. Significant correlations are in bold (*p* < .05 following Bonferroni alpha correction).

|  | 1. | 2. | 3. | 4. | 5. | 6. | 7. | 8. | 9. | 10. |
| --- | --- | --- | --- | --- | --- | --- | --- | --- | --- | --- |
| 1. Gender | – | **-.22** | .03 | .09 | .06 | -.01 | -.02 | .03 | .06 | .05 |
| 2. Age | **-.14** | – | .04 | .02 | -.04 | -.07 | -.11 | **-.17** | .05 | .05 |
| 3. English Proficiency | .05 | .02 | – | .08 | -.07 | .10 | **-.21** | -.13 | .09 | .07 |
| 4. NFC | .08 | .02 | .10 | – | **-.16** | .12 | **-.18** | **-.32** | **.94** | **.21** |
| 5. Random IER | .04 | -.03 | -.04 | **-.13** | – | **-.21** | .13 | -.15 | **-.23** | **.52** |
| 6. Non-Random IER | -.03 | -.08 | .06 | **.13** | **-.21** | – | **-.30** | -.05 | **.18** | -.07 |
| 7. Direct IER Total | .03 | -.03 | **-.23** | **-.27** | .05 | **-.22** | – | **.26** | **-.27** | -.09 |
| 8. IRTree Midpoint | -.05 | **-.12** | -.09 | **-.20** | -.08 | -.10 | .06 | – | **-.27** | **-.60** |
| 9. IRTree Agreement | .04 | .01 | .09 | **.85** | -.06 | **.16** | **-.39** | -.06 | – | .09 |
| 10. IRTree Extreme | .10 | .07 | .05 | **.12** | **.49** | -.04 | .08 | **-.58** | -.06 | – |

*Note.* Correlations for data prior to cleaning are below the diagonal, correlations after cleaning are above the diagonal. Uncleaned *n* = 740-741; cleaned *n* = 406-407. Significant correlations are in bold (*p* < .05 following Bonferroni alpha correction).
